# Unravelling carbon monoxide protection in cerebral ischemia: from the organelle to the organism

**DOI:** 10.1186/2193-1801-4-S1-L26

**Published:** 2015-06-12

**Authors:** Claudia Queiroga, Ana Almeida, Simone Tomasi, Alessandro Vercelli, Paula Paula, Helena Vieira

**Affiliations:** Chronic Diseases Research Center, Universidade Nova de Lisboa, Portugal; Neuroscience Institute Cavalieri Ottolenghi, Italy; Instituto de Biologia Experimental e Tecnológica/Instituto de Tecnologia Química e Biológica, Universidade Nova de Lisboa, Portugal

**Keywords:** Carbon monoxide, preconditioning, ischemia

Perinatal complications are a serious clinical problem, in particular hypoxic-ischemic (HI) episodes, caused by birth asphyxia or uterine and fetal blood flow interruption. HI corresponds to 23% of neonatal deaths, being one of the top 20 leading causes of disease burden. Preconditioning (PC) is a stimulation below the injury threshold that activates endogenous protective mechanisms to prevent damage. Low doses of carbon monoxide (CO) play a beneficial role through PC induction. Herein, CO cytoprotection was explored in distinct brain models. The used experimental models range from monoculture of astrocytes, co-cultures of neurons and astrocytes, to the whole organism with the rat model of perinatal ischemia. In primary cultures of astrocytic cells, CO not only impairs mitochondrial membrane permeabilization, by ANT glutathionylation, but also strengths mitochondrial oxidative metabolism, by modulating COX activity, increasing mitochondrial biogenesis and ATP amounts. Also, CO reinforces astrocytes-neurons communication towards neuronal survival. Purinergic molecules are the main mediators for this non-cell autonomous effect. Our results seem to indicate that the main pathway involved includes ATP release from astrocytes, its metabolization and A2A receptor binding to initiate protective mechanisms within the neurons. Rat pups were exposed to CO before hypoxia-ischemia induction (Vannucci model). 24h after HI the brains were collected for cell death and tissue protection assessment (histological and immunohistochemical analysis). It was found limited apoptosis in hippocampus following cerebral ischemia: lower cytochrome c release and caspase-3 activation yielding an increased Bcl-2 expression. Altogether, one can conclude that there is not just a unique pathway for the CO-induced endogenous protection, brain tolerance is the result of a complex cellular change in response to injury. Indeed, CO regulates cell death pathways and modulates cellular metabolism.

